# Regulation of mRNA stability contributes to the function of innate lymphoid cells in various diseases

**DOI:** 10.3389/fimmu.2023.1118483

**Published:** 2023-01-26

**Authors:** Yuanyu Deng, Saiyu Shi, Jie Luo, Yiwei Zhang, Hui Dong, Xian Wang, Jian Zhou, Zhiyuan Wei, Jiahui Li, Chen Xu, Shuai Xu, Yi Sun, Bing Ni, Yuzhang Wu, Di Yang, Chao Han, Yi Tian

**Affiliations:** ^1^ Institute of Immunology, PLA, Third Military Medical University (Army Medical University), Chongqing, China; ^2^ Institute of Pathology and Southwest Cancer Center, Southwest Hospital, Third Military Medical University (Army Medical University), Chongqing, China; ^3^ Department of Immunology, Medical College of Qingdao University, Qingdao, Shandong, China; ^4^ The First Affiliated Hospital of Third Military Medical University (Army Medical University), Chongqing, China; ^5^ Department of Stomatology, Xinqiao Hospital, Third Military Medical University (Army Medical University), Chongqing, China; ^6^ Department of Pathophysiology, Third Military Medical University (Army Medical University), Chongqing, China

**Keywords:** ILCs, mRNA stability, cytokines, RBPs, non-coding RNA

## Abstract

Innate lymphoid cells (ILCs) are important subsets of innate immune cells that regulate mucosal immunity. ILCs include natural killer cells, innate lymphoid cells-1 (ILC1s), ILC2s, and ILC3s, which have extremely important roles in the immune system. In this review, we summarize the regulation of mRNA stability mediated through various factors in ILCs (e.g., cytokines, RNA-binding proteins, non-coding RNAs) and their roles in mediating functions in different ILC subsets. In addition, we discuss potential therapeutic targets for diseases such as chronic obstructive pulmonary disease, cancer, and pulmonary fibrosis by regulation of mRNA stability in ILCs, which may provide novel directions for future clinical research.

## 1 Introduction

Innate lymphoid cells (ILCs) are located throughout the human body. Most ILCs are enriched in mucosal structures, such as skin, lung, and digestive tract, and many ILCs are found in adipose tissues and lymph nodes. ILCs play an important part in regulating immune balance in tissues, resisting pathogenic infection, and enhancing adaptive immunity (1–3). Inappropriate activation of ILCs is associated with chronic obstructive pulmonary disease, cancer, and pulmonary fibrosis (4, 5).

For several decades, the discovery and functional studies of ILCs have enhanced understanding of immune regulation in mucosal tissues considerably. However, the development process and phylogenetic classification of ILC subsets are controversial.

ILCs are divided into two cell types: cytotoxic ILCs (which mainly represent conventional natural killer (NK) cells) and helper-like ILCs, which produce cytokines (e.g., innate lymphoid cells-1 (ILC1s), innate lymphoid cells-2 (ILC2s), innate lymphoid cells-3 (ILC3s)) (4).

NK cells and ILC1s are considered to belong in the same group of ILCs due to their secretion of interferon-γ (IFN-γ) and tumor necrosis factor-α (TNF-α) (6). Studies have shown that ILC1s cannot control the growth or metastasis of local tumors, whereas NK cells favor tumor monitoring, possibly because transforming growth factor-β (TGF-β) signaling can convert NK cells into intermediate-ILC1s and ILC1s in the tumor microenvironment to drive immune system evasion (7, 8).

The lineage-specific transcription factor GATA binding protein-3 (GATA-3) shows high expression in ILC2s and mediates multiple functions by producing various cytokines (e.g., interleukin-4 (IL-4), IL-5, IL-9), and amphiregulin. Specifically, type-2 cytokines derived from ILC2s play a crucial part in preventing parasitic infection and inducing eosinophilic inflammation, which participates in asthma, eosinophilic esophagitis, and chronic rhinosinusitis (9).

ILC3s are a heterogeneous cell population. They include natural cytotoxic receptor (NCR) cells, which consist of lymphoid tissue inducer (LTi) cells and LTi-like cells, as well as NCR^+^ ILC3s. Nuclear hormone retinoic acid receptor-related orphan receptor γt (ROR γt) is required for the development and function of ILC3s, similar to T-helper type 17 (Th17) and Th22 cells (10).

Cytokines and cytokine receptors are pivotal for the function of ILCs. TGF-β acts on its receptor TGF-β receptor 2 (TGF-βR2) and attenuates IFN-γ secretion by ILC1s (11). Type-I interferons, IL-2, IL-12, IL-15, IL-18, IL-21 and their receptors positively regulate NK-cell function, whereas the interaction of IL-23, IL-27 and their receptors, may suppress or enhance NK-cell function (12). ILC2s show high expression of IL-7 receptor α (IL-7Rα), IL-33 receptor (IL-33R) and IL-17 receptor β (IL-17Rβ) (13). Therefore, epithelial cell-derived thymic stromal lymphopoietin (TSLP), IL-33 and IL-25 can stimulate ILC2s to produce various type-2 cytokines (14). ILC3s and LTi, which express IL-23 and IL-1β receptor (IL-1βR), are stimulated by IL-23 and IL-1β to produce the signature cytokines IL-17 and IL-22 (15).

A growing body of research suggests that key mechanisms of gene regulation, including transcriptional and posttranscriptional regulation, have vital roles in the phenotype and function of ILCs. Transcriptional regulation has been studied thoroughly, but posttranscriptional regulation is much less explored. Posttranscriptional regulation is reflected mainly in the: splicing and processing of mRNA precursor heterogeneous nuclear RNA; processing and localization of mRNA from the nucleus to cytoplasm; stability or degradation of mRNA in cytoplasm. RNA editing and RNA interference also belong to the category of posttranscriptional regulation (16–20).

In recent years, several studies have revealed that mRNA stability affects the function of ILCs (21, 22). The first mechanism of mRNA degradation is exonucleolytic degradation, which removes the poly(A) tail (PAT) by exonucleases, such as the carbon catabolite repression 4 (CCR4)-negative on TATA-less (NOT) complex or poly(A)-specific ribonuclease (PARN). mRNA can be degraded from either the 3’ end or 5’ end. Degradation from the 3’ end is associated with the binding of exosomes containing exonucleases. Degradation from the 5’ end is initiated by the formation of a complex at the 3’ end that recruits a decapping enzyme to remove the 5’ cap, and then the mRNA is degraded by 5’→3’ exonuclease. The second mechanism of mRNA degradation is endonucleolytic cleavage. For example, site-specific ribonucleases can induce internal cleavage to produce RNA fragments, which are then degraded by exonucleases. The third mechanism of mRNA degradation is nonsense-mediated degradation (NMD), which inhibits the production of aberrant proteins by eliminating abnormal mRNAs with premature termination codons (PTCs). PTCs are recognized by a protein complex that contains up-frameshift proteins during the progression of translation, which stalls the subsequent translation and recruits degradation machinery (23, 24).

Next, we focus on the role of mRNA stability regulation in the three major ILC subsets in various diseases.

## 2 Regulation of mRNA stability can affect the antiviral, immunoregulatory, and antitumor properties of NK cells

ILC1s and NK cells mainly work together in protective responses to intracellular bacteria and viruses and in cancer immunosuppression. However, the phenotypic maturation of NK cells is dependent on the transcription factor eomesodermin (Eomes), which is highly cytotoxic (25). Under inflammatory conditions, upregulation of stress ligands in host cells can trigger activation of NK cells. In contrast, the deficiency of major histocompatibility complex type-I (MHC-I) molecules during viral infection induces inhibition of NK-cell activity (26, 27). The mRNA stability of IFN-γ, IL-10, urokinase plasminogen activator (uPA), and the receptor of uPA (uPAR) in NK cells can be regulated by many stimulating factors, such as cytokines and pathogens (**Table 1**).

**Table 1:** Regulation of mRNA stability in ILCs is involved in various diseases.

ILC subset	Factors		Target mRNA	Stability	Function	References
NK cells	Cytokines	TGF-β, IL-18	IFN-γ mRNA	Destabilization	Prevent immune dysregulation and diseases	(28, 29)
		IL-2, IL-12		Stabilization	Modulate immunoregulatory properties	(30, 31)
		IL-33		Stabilization	Affect antiviral properties	(32)
		IL-12, IL-18		Stabilization	Regulate immunity against infectious pathogens	(33)
		IL-2	uPA, uPAR mRNA	Stabilization	Affect antitumor properties	(34)
	Pathogen	*Leishmania* species	IL-10 mRNA	Stabilization	Inhibit host resistance to an intracellular pathogen	(35)
ILC2	RBPs	Regnase-1	Egr1, ICOS mRNA	Destabilization	Inhibit pulmonary fibrosis	(36)
		TTP	IL-5 mRNA	Destabilization	Regulate intestinal homeostasis	(37)
	miRNA	miRNA-155	c-Maf mRNA	Destabilization	Inhibit allergic rhinitis	(38)
		miRNA-155-5p	TP53INP1 mRNA	Destabilization		(39)
ILC3	CircRNA	circZbtb20	Nr4a1 mRNA	Stabilization	Decrease susceptibilities to bacterial infection	(40)

### 2.1 Cytokines regulate the stability of IFN-γ mRNA through protein kinase C and p38 mitogen-activated protein kinases signaling pathways in NK cells

**Figure 1:**
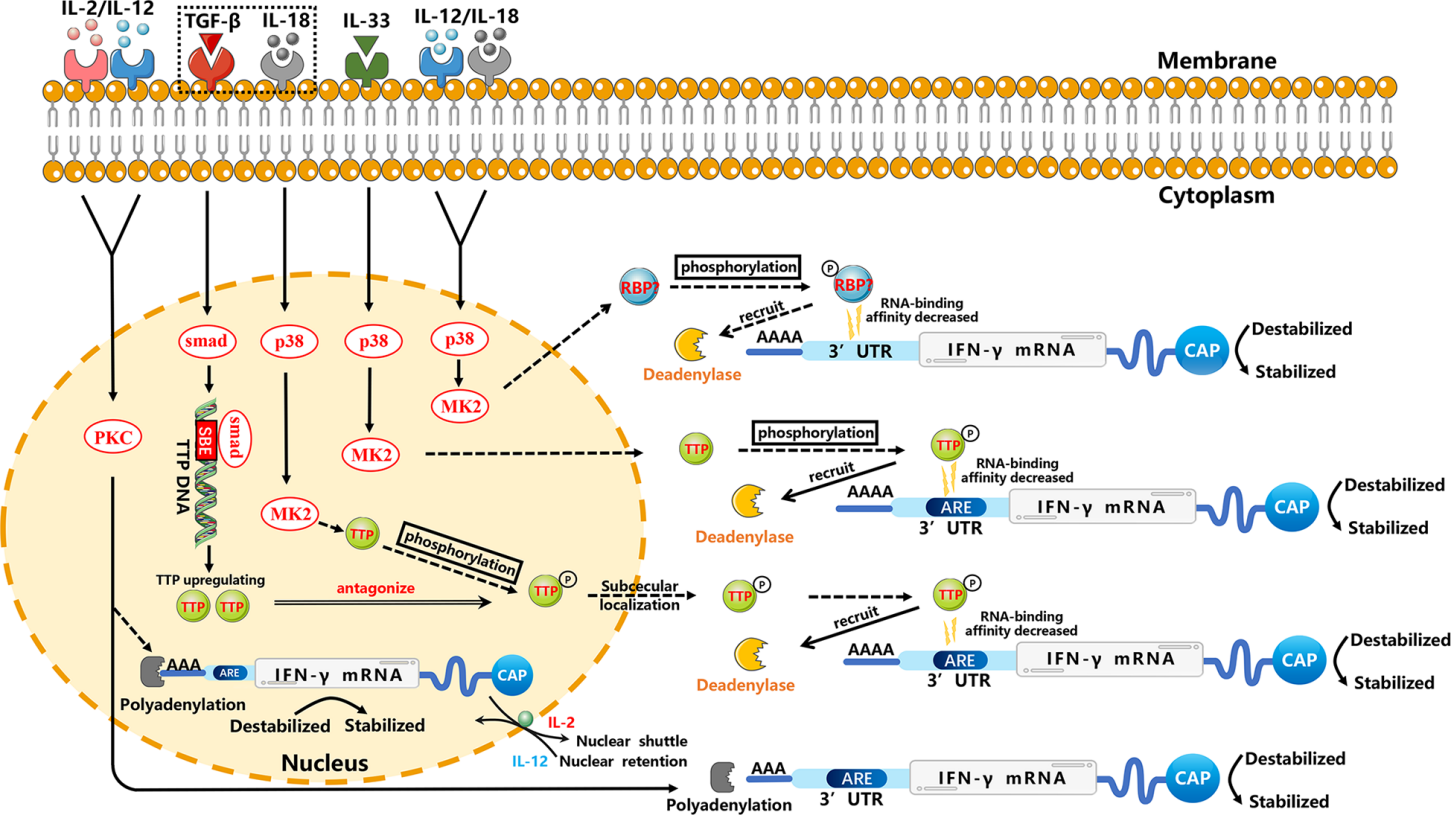
Regulation of the stability of IFN-γ mRNA in NK cells through p38 MAPK and PKC signal-transduction pathways. For clarity, these posttranscriptional regulators are not necessarily placed at the correct cell site. IL-12 and IL-18 can enhance the stability of IFN-γ mRNA in NK cells through the p38 MAPK pathway, which may phosphorylate unknown RBPs to result in decreased RNA-binding affinity and then decreased deadenylation. TGF-β inhibits the stability of IL-18-induced IFN-γ mRNA. IL-18 may inhibit TTP mRNA and affect the stability of IFN-γ mRNA through the p38 MAPK pathway, which may phosphorylate TTP. This phosphorylation results in the decreased RNA-binding affinity of TTP, which can trigger deadenylation of IFN-γ mRNA by recruiting cytoplasmic deadenylase. However, TGF-β can upregulate TTP transcription specifically through the Smad pathway. Smad proteins downstream of TGF-β receptors can mediate a stimulatory effect by binding the SBE of the TTP promoter (45), thereby repressing expression of IL-18-induced IFN-γ mRNA through TTP-induced destabilization. A similar TTP-related mechanism may be found in IL-33-stimulated NK cells. IL-2 and IL-12 may affect the stability of cytoplasmic IFN-γ mRNA and stability of the nuclear-processed form of IFN-γ mRNA through PKC-dependent polyadenylation in NK cells. In addition, IL-12 mediates nuclear shuttling whereas IL-2 mediates nuclear retention, which may be related to AREs, but the specific mechanism requires further investigation. Solid arrows indicate confirmed mechanisms. Dashed arrows indicate possible mechanisms that need further verification. Molecular interactions and all abbreviations are explained in the text.

IFN-γ plays a vital part in regulating the immune response. IFN-γ produced by NK cells has antiviral, immunoregulatory, and antitumor properties (41, 42). The antitumor function of NK cells mediated by IFN-γ can induce apoptosis, tumor dormancy, and immunoediting in tumor cells that are related to the relapse and progression of tumors (43, 44). Recently, several studies have shown that PKC and p38 MAPK signaling pathways have critical roles in the cytokine-mediated stability of IFN-γ mRNA (**Figure 1**).

Many studies have shown that p38 MAPK is activated through different ways to regulate the mRNA stability of various cytokines. This regulation involves mRNA elements (e.g., adenylate-uridylate (AU)-rich elements (AREs)) and mRNA-binding proteins (46). The effect of AREs on mRNA stability relies on moieties which selectively recognize these elements and regulate the fate of mRNA *via* interacting with the machineries related to regulation of mRNA stability. The p38 MAPK signal-dependent phosphorylation of ARE-binding protein affects its binding to AREs and prevents degradation by recruiting the deadenylase PARN, exosome, and decapping enzyme (46). Often, the mRNA-binding protein phosphorylation affects the RNA-binding affinity or subcellular localization. For example, phosphorylated KSRP (a vital factor for ARE-mediated mRNA decay) on T692 by p38 MAPK reduces its affinity toward AREs, resulting in increased stability of transcripts containing ARE (47). Also, MK2 (a kinase downstream of p38 MAPK) mediates phosphorylation at serine 52 and serine 178 to promote redistribution of zinc finger protein 36 (TTP) from nucleus to cytoplasm in HeLa cells (48). Furthermore, the phosphorylation of mRNA-binding proteins can alter interaction among mRNA-binding proteins and change scaffolding properties for mRNA-modifying enzymes, such as polyadenylases, deadenylases, poly (A) binding proteins (PABP), decapping enzymes, endonucleases, exonucleases, and exosome proteins. In many cases, the phenomenon results in mRNA stabilization (46). In addition to AREs, the p38 MAPK signaling pathway can regulate mRNA stability through the 3′ untranslated region (3′UTR), which has a critical role in regulation of mRNA stability (49).

#### 2.1.1 IL-12 and IL-18

IL-12, produced primarily by monocytes and macrophages, stimulates the growth of NK cells (at least in part) and Th1 cells through binding to its receptor on these cells (50). The effect elicited by IL-18 resembles the effect of IL-12, possibly because they synergize (51). In NK cells stimulated by IL-12 plus IL-18, IFN-γ mRNA was shown to be destabilized potently by a specific inhibitor of p38 MAPK, and its half-life decreased, which demonstrated that IL-12 plus IL-18 could enhance the stability of IFN-γ mRNA rapidly through the p38 MAPK signaling pathway and, thus, increase the expression of IFN-γ in NK cells (33). Simultaneously, destabilization of IFN-γ mRNA by inhibitors of p38 was accompanied by shortening of the PAT (52), thereby illustrating that regulation of the stability of IFN-γ mRNA might be related to the PAT. Some studies have shown that IL-12- plus IL-18-induced IFN-γ stabilization might be caused by blocking of mRNA deadenylation (52–54), but the specific RBP acting on the IFN-γ mRNA has not been identified.

#### 2.1.2 IL-33

IL-33, a nuclear cytokine from the IL-1 family, is expressed abundantly in epithelial, endothelial, and fibroblast-like cells (55). Upon tissue damage, it acts as an alarm signal (alarmin) to alert immune cells expressing IL-1 receptor like 1 (IL-1RL1), such as tissue-resident immunological cells, including regulatory T cells (Tregs), mast cells, ILC2s, NK cells and macrophages (55). Ochayon et al. measured the phosphorylation and downstream targets of p38 MAPK in NK cells through flow cytometry (FCM) and treated the culture with actinomycin D to inhibit production of nascent RNA, thereby permitting the decay measurement of existing IFN-γ transcripts in NK cells. They revealed that IL-33 could enhance the mRNA stability of IFN-γ and then promote IFN-γ expression, which was mediated by rapid induction of phosphorylation of the activated protein-1-dependent p38 MAPK signaling pathway (32). Posttranscriptional regulation of cytokines by MK2/3 in macrophages was mediated by TTP, which could regulate the stability of AREs containing mRNAs (56). We have hypothesized analogous underlying mechanisms in NK cells, which need to be investigated further.

MK2 phosphorylation regulates inflammation (57, 58). However, only a partial inhibitory effect on IL-12/IL-33 synergistic interactions after suppressing MK2 has been noted compared with the inhibition of p38 MAPK (32). These findings imply that other p38 MAPK-induced mediators, such as activation transcription factor 2 (ATF2), may also be critical for the enhancement of IL-33 effect, and additional studies focusing on correlation with regulation of mRNA stability are needed (32).

#### 2.1.3 TGF-β and IL-18

TGF-β has three known family members (TGF-β1, TGF-β2 and TGF-β3) in mammals that can regulate various physiological processes (59). Inoue et al. reported that TGF-β1 inhibited IL-18-induced IFN-γ production and antiviral activity by downregulating the stability of IFN-γ mRNA in a mouse line of NK cells (LNK5E6) (28). They also found that the IFN-γ mRNA stability was regulated by destabilizing elements in the IFN-γ mRNA 3′UTR, particularly AREs in 5’ half of the 3′UTR (29). However, the underlying mechanism of stability regulation is incompletely understood. A similar mechanism has been reported in a human myelomonocytic cell line (KG-1). IL-18 inhibited TTP mRNA and led TTP phosphorylation by inducing p38 MAPK-activated MK2, and then localized TTP to the cytoplasm to interact with ARE. Phosphorylated TTP lost its ability which could trigger the deadenylation of IFN-γ mRNA due to blockade of the recruitment of cytoplasmic deadenylases (60, 61). However, TGF-β could induce multiple cell types to upregulate TTP transcription through the Smad pathway. Smad proteins, downstream of TGF-βRs, can mediate a stimulatory effect by binding to the Smad-binding element (SBE) of the TTP promoter (45), thereby repressing IL-18-induced IFN-γ mRNA expression through TTP-induced destabilization (62, 63).

#### 2.1.4 IL-2 and IL-12

IL-2 and IL-12 strongly regulate IFN-γ expression (30, 31). Ye et al. demonstrated that in NK3.3 cells (a human NK cell line dependent on IL-2), the increase of IFN-γ protein induced by stimulation of IL-2 plus IL-12 was due largely to the enhanced stability of cytoplasmic IFN-γ mRNA. Additional studies revealed that IFN-γ mRNA induced by IL-2 plus IL-12 in NK cells required PKC activation, showing that regulation of the stability of IFN-γ mRNA by IL-2 plus IL-12 might be mediated by the PKC pathway (30). Hodge et al. showed a novel manner of posttranscriptional regulation in IFN-γ expression: the role of IL-2, IL-12 and polyadenylation was related to mRNA stability (31, 64–67). IL-2 plus IL-12 appeared to make the processed form of mRNA more stable, and then promoted increased nucleocytoplasmic shuttling of mature IFN-γ mRNA (31). However, compared with IL-2- plus IL-12-induced cytoplasmic stabilization, IL-12 had nuclear effects which resulted in increased accumulation of the precursor and processed IFN-γ mRNA. Interestingly, IL-2 could overcome the IL-12-induced nuclear-retention of IFN-γ mRNA, which resulted in rapid shuttling of IFN-γ mRNA. In other words, the IL-12-induced nuclear retention of IFN-γ mRNA prevailed until receiving IL-2 stimulation, which mediated transcription-independent movement of nuclear IFN-γ mRNA. Ultimately, the increased expression of IFN-γ mRNA in cytoplasm resulted in the enhanced synthesis and secretion of IFN-γ protein from NK92 cells (31). Nuclear retention of IFN-γ mRNA might be mediated by a specific mRNA element, such as the AREs of IFN-γ mRNA in its 3′UTR. This speculation was supported by the observation that a sequence-mediated nuclear mRNA-retention element (which was identified in the U2AF mRNA) could prevent transport of nucleocytoplasmic mRNA (68). However, the role of AREs in mRNA nuclear retention has not yet been determined, and needs further investigation.

### 2.2 Infection with *Leishmania* parasites alters the stability of IL-10 mRNA to affect anti-inflammation mechanisms in NK cells

IL-10 is an anti-inflammatory cytokine and its expression is controlled by some transcription factors, such as specificity protein 1 (Sp-1) and Sp-3 (69). IL-10 can contribute to the development of many diseases (70–72). NK cells can also differentiate to produce IL-10 and may have regulatory activity (73). IL-10 mRNA 3 ‘UTR has multiple copies of the potential mRNA destabilization motif (AUUUA), which are associated with the regulation of mRNA stability and located in three potential regulatory regions (AU1, AU2, AU3) (74, 75). Maroof et al. described a posttranscriptional mechanism of regulation involving *IL-10* expression that induced long-term activation *in vivo* after infection with *Leishmania* parasites and enhanced stability of IL-10 mRNA in NK cells. Those actions enhanced the production of IL-10 protein and the inhibitory function of NK cells to diminish host resistance to an intracellular pathogen (35). The specific regulatory mechanism of stability of IL-10 mRNA has not been reported in NK cells, but a precedent has been found in macrophages that control IL-10 secretion. Specifically, upon macrophage activation, IL-10 transcription was upregulated through phorbol 12-myristate 13-acetate response elements located in the promoter of *IL-10*. Then, the degradation of IL-10 mRNA was inhibited, which was related to these three regulatory regions in the IL-10 mRNA 3’UTR (74, 75). Recently, several ARE-binding proteins (76–80) and ARE-recognizing proteasomes (81) have been reported, which might be involved in the destabilization of IL-10 mRNA through regulation of AU2 in macrophages. However, the molecular mechanism underlying infection by *Leishmania* species-induced enhancement of the stability of IL-10 mRNA in NK cells is not clear, and additional studies are needed to determine if similar mechanisms exist in NK cells.

### 2.3 IL-2 modulates the accumulation, metastasis, and cytotoxicity of NK cells by regulating the stability of uPA/uPAR mRNA in NK cells

uPA and uPAR have major roles in NK cell-mediated decay of the extracellular matrix (82, 83). These roles may be related to the accumulation, metastasis, and cytotoxicity of NK cells (84). Recently, *via* Matrigel™-based studies, an increase in the number of invading NK cells has been reported after IL-2 stimulation, and this invasion has been demonstrated to employ the uPA system (84, 85). Al-Atrash et al. revealed that IL-2 upregulated expression of uPA and uPAR in NK cells, and that the half-life of uPA mRNA in IL-2-treated cells was slightly longer than that in unstimulated cells. Therefore, the enhanced stability of uPA mRNA might be a contributory factor to its expression upregulation (34). In lung fibroblasts as well as in epithelial, carcinoma, and mesothelial cells, the expression regulation of uPA and uPAR has been shown to occur at a posttranscriptional level, which was mediated by the interaction of a 30-kD RBP to the uPA mRNA 3′UTR (86) and a 50-kD RBP to the coding region of uPAR mRNA (87–89), which resulted in destabilization of the binding of uPA/uPAR mRNA. Moreover, the expression of uPAR RBP decreased, which might contribute to the increase of uPAR mRNA after IL-2 treatment in YT cells (a NK-like lymphoid cell line). However, other mechanisms cannot be excluded, such as structural modification of uPAR RBP. Therefore, Al-Atrash et al. indicated that the mRNA stability of uPAR in IL-2-stimulated YT cells appeared to be regulated (at least partly) by destabilizing RBPs interactions. Under such conditions, modifying the stability of uPA mRNA in NK cells could enhance the infiltration of NK cells into tumors, thus destroying tumors more effectively (90–93). Therefore, increasing invasiveness by NK cells before administration of NK cells in cancer immunotherapy by upregulating regulation of uPA and/or uPAR expression may increase infiltration into tumors and eradication of tumor cells by NK cells.

## 3 Regulation of mRNA stability can affect pulmonary fibrosis, allergic rhinitis, and the intestinal homeostasis of ILC2s

ILC2s play a vital part in preventing parasitic infection and inducing eosinophil inflammation by secreting type-2 cytokines. ILC2s are involved in regulating intestinal homeostasis and the development of diseases, such as pulmonary fibrosis and allergic rhinitis (94–99). In addition, an increasing number of studies have revealed that RBPs and microRNAs (miRNAs) have crucial roles in regulating the homeostasis of ILC2s through control of mRNA stability.

The next part of this review focuses on the notion that mRNA stability of early growth response 1 (Egr1), inducible costimulator (ICOS), IL-5, c-Maf and tumor protein p53-induced nuclear protein (TP53INP1) in ILC2s can be regulated by RBPs and miRNAs (**Table 1**).

### 3.1 RBPs maintain ILC2s homeostasis by regulating the mRNA stability of Egr1, ICOS, and IL-5

#### 3.1.1 Regnase-1

Regnase-1 encoded by MCPIP1 (ZC3H12A) inhibits gene expression through mRNA degradation (100, 101). Yoshinari et al. screened the binding sites of transcription factors for upregulated genes related to “pulmonary fibrosis” in Comparative Toxicogenomics Database. They identified a set of transcription factors that may regulate gene expressions associated with pulmonary fibrosis. Among the identified transcription factors, regnase-1 deficiency resulted in >60-fold higher expression of Egr1 (which enhances the transcription of various fibrosis-associated genes (102, 103)) in ILC2s. The overexpression of wild-type (WT) regnase-1 rather than mutated regnase-1 inhibited the luciferase reporter harboring the 3′UTR of Egr1, suggesting that the mRNA of Egr1 was degraded by regnase-1. In addition to Egr1, ICOS has been reported that it isto be positively associated with the amount of ILC2s in the blood of patients with idiopathic pulmonary fibrosis (36). Maazi et al. found that ICOS had an important role in the survival of ILC2s and cytokine production through the IL-2 and signal transducer and activator of transcription 5 (STAT5) pathway (104). Yoshinari et al. found that ICOS expression was upregulated significantly in ILC2s deficient in regnase-1 (36). Uehata et al. showed that regnase-1 degraded ICOS mRNA directly through the 3′UTR in T cells (105): additional studies needed to be ascertained if similar mechanisms exist in ILC2s. Taken together, those data suggested that the posttranscriptional regulation of Egr1 and ICOS by regnase-1 in ILC2s suppressed the pro-fibrotic function of ILC2s in lung. Therefore, regnase-1 is regarded as a crucial posttranscriptional regulator for the pro-fibrotic function of ILC2s in mice and humans, and its regulatory mechanism for Egr1 and ICOS may provide novel therapeutic strategies for idiopathic pulmonary fibrosis. However, further studies are needed of all the transcriptional and posttranscriptional gene-regulation networks that can regulate pulmonary fibrosis by ILC2s (36).

#### 3.1.2 TTP

The tristetraprolin family, which includes TTP, zinc finger protein 36 like 1, and zinc finger protein 36 like 2, has critical roles in posttranscriptional regulation, especially for regulation of mRNA stability (106). TTP (also known as TIS11, Zfp36, and Nup475) contains two zinc-finger domains that have a crucial role in the posttranscriptional regulation of inflammation (107–109). Hikichi et al. overexpressed TTP significantly using a retroviral vector and found that the production of IL-5 was inhibited in IL-2- and IL-33-stimulated ILC2s. They assessed the stability of IL-5 mRNA and found that TTP overexpression could enhance mRNA degradation. Those results suggested that TTP regulated IL-5 expression in ILC2s through mRNA degradation (37). Hikichi et al. undertook luciferase reporter assays with IL-5 3′UTRs. They created three constructs through mutation of different AREs in the IL-5 3′UTR and carried out a reporter experiment. Those results showed that TTP regulated IL-5 expression directly by binding to the IL-5 mRNA △ ARE 5-7 sequence, which led to a reduction in the stability and expression of IL-5 mRNA. Studies have demonstrated that the mechanism of mRNA degradation by TTP involves the C-terminal domain recruiting a deadenylase to remove the PAT (109).

IL-5, derived from ILC2s, has been reported to maintain steady-state eosinophils in small intestine (110). Hence, the mucosal barrier can be protected by these homeostatic eosinophils in the small intestine through supporting immunoglobulin-A secretion from plasma cells (111, 112). Hikichi et al. analyzed the eosinophil number in small intestine and lungs of Zfp36-deficient mice. In accordance with the increased number of ILC2s, the accumulation of eosinophils was significantly enhanced in small intestine of Zfp36-deficient mice. Those results indicated that TTP regulated eosinophil number *via* an appropriate production of ILC2s-derived IL-5, thereby contributing to intestinal homeostasis (37).

### 3.2 miRNA affects allergic rhinitis by regulating the stability of c-Maf mRNA and TP53INP1 mRNA in ILC2s

miRNAs, as one of small and noncoding RNAs, regulate gene expression at posttranscriptional level specifically (113). miRNAs can load into the RNA-induced silencing complex (RISC) and then bind to the 3′UTR of target mRNAs, which results in the degradation or translational inhibition of mRNA (114).

miRNA-155 is a key regulator for ILC2s and NK cells, impacting their development and functions (115–117). Through database analysis with bioinformatics software (TargetScan, PicTar, miRanda) and luciferase reporter assays, Zhu et al. suggested that miRNA-155 targeted WT c-Maf 3′UTR. Using reverse transcription-quantitative polymerase chain reaction (RT-qPCR) and enzyme-linked immunosorbent assay (ELISA), Zhu et al. determined the roles of miRNA-155 in the expression of c-Maf and Th2 cytokines in ILC2s. miRNA-155 overexpression inhibited the expression of c-Maf mRNA and protein significantly, and increased Th2 cytokines expression including IL-4, IL-5, IL-9 and IL-13, in ILC2s. Those results indicated that miRNA-155 decreased the mRNA and protein expression of c-Maf and promoted expression of Th2 cytokines in ILC2s by binding to the c-Maf mRNA 3′UTR (38). In allergic rhinitis, increased activity of ILC2s and release of Th2 cytokines have been considered to be the main causes of disease aggravation (38). Reduced c-Maf expression in the nasal mucosa of mice and patients with allergic rhinitis was recovered significantly by administration of miRNA-155 antagomir, suggesting that miRNA-155 might be a therapeutic target for allergic diseases (38). However, whether miRNA-155 affects the secretion of Th2 cytokines in ILC2s through regulating the stability of c-Maf mRNA is not known.

Zhu et al. demonstrated that there was a target RNA of miRNA-155-5p in ILC2s: TP53INP1 (a tumor-suppressor protein). miRNA-155-5p could downregulate the stability of TP53INP1 mRNA and expression of TP53INP1 through binding to a specific sequence (AGCAUUAA) in TP53INP1 mRNA 3’UTR. TP53INP1 is negatively correlated with miRNA-155-5p. In contrast, TP53INP1 could inhibit the production of Th2 cytokines in ILC2s and promote the production of Th1 cytokines and ILC2s apoptosis. miRNA-155-5p showed high expression in allergic rhinitis. Studies have demonstrated that TP53INP1 may have an anti-inflammatory role because it can inhibit the nuclear factor-κ B (NF-κB) signaling pathway in patients with allergic rhinitis, but the specific mechanism is largely unknown (39).

## 4 Regulation of mRNA stability can modulate susceptibilities to bacterial infection of ILC3s

ILC3s have critical roles in innate immunity and gut homeostasis (118). In recent years, mRNA stability in ILC3s has been reported to be regulated by circular RNAs (circRNAs). The section below provides evidence that the stability of Nr4a1 mRNA in ILC3s can be regulated by circRNAs (**Table 1**).

### 4.1 CircRNAs maintain ILC3s homeostasis by regulating the stability of Nr4a1 mRNA

circRNAs are a class of non-coding RNAs with a ring structure. They are formed by spliceosomes or nested splicing (119). Studies have shown that circRNAs can be formed in ILC3s-induced intestinal inflammation (120) and enhance the ability to resist bacterial infection (121). Liu et al. identified a circRNA (circZbtb20) through a direct interaction with the RNA pulldown assay and sequence alignment, which showed high expression of circZbtb20 in ILC3s. circZbtb20 maintained the homeostasis and function of ILC3s by enhancing the stability of Nr4a1 mRNA. Increasing numbers of studies have revealed a close correlation between Nr4a1 and regulation of the immune response. Also, Nr4a1 can initiate transcription of Notch2 to promote its expression, which is required for maintenance of the expansion and function of ILC3s. Liu et al. used the biotin-labeled linearized circZbtb20 as “bait” to conduct RNA-pulldown experiments. They found that circZbtb20 could enhance the interaction between Nr4a1 mRNA and AlkB homolog 5 (Alkbh5) (40). Alkbh5 is a demethylase for N6-methyladenosine (m6A)-modification, and is related to the mRNA stability regulation (122). M6A regulates the degradation of methylated RNA and is negatively correlated with mRNA stability (123). Liu et al. found that Alkbh5 deficiency increased m6A modification of Nr4a1 mRNA in ILC3s using single-base elongation- and ligation-based qPCR (40). Liu et al. demonstrated that the circZbtb20–Alkbh5–Nr4a1–Notch2 axis was important for the homeostasis and function regulation of ILC3s, and that the destruction of this axis increased susceptibilities to bacterial infection (40).

## 5 Conclusions and perspectives

ILCs are a fascinating cell population and have important roles in several diseases. In the past decade, there has been an explosion of knowledge surrounding the identification, development plans, and functionality of ILCs.

Here, we summarized the current findings of the regulation of mRNA stability on ILCs function. In particular, we focused on the effects of RBP on regulation of mRNA stability in ILCs. First, even though multiple cytokines (e.g., IL-12, IL-18) have been shown to have important regulatory roles on ILC1s function, mRNA stability-related RBPs in ILC1s have not been identified, which merits further investigation. Second, several RBPs have been shown to have important roles in the regulation of ILC2s function, including regnase-1, TTP, and RNA-binding protein 3 (RBM3) (124). Specifically, regnase-1 and TTP have broad roles in mRNA stability in ILCs and other cell subsets. However, RBM3 has a potentially specific role in ILC2s because studies have shown that RBM3 deficiency has no effects on the numbers of NK cells, T cells, or B cells in mice. Hence, RBM3 might be an ILC2s-specific RBP in immune cells (124). Third, a few studies have reported that circRNAs can regulate downstream mRNAs stability in ILC3s indirectly (125–127). Therefore, researchers need to demonstrate the roles of mRNA stability on the regulation of ILC3s function. Taken together, evidence suggests a novel molecular mechanism for the regulation of ILCs functions, which may represent a new intervention for ILCs-related diseases such as idiopathic pulmonary fibrosis, allergic rhinitis, and cancer.

## Author contributions

Author contribution: YT, CH, DY, YW and BN: Conceived and supervised this study. YD and SS: Wrote the manuscript. JL, YZ, HD, XW, JZ, ZW, JHL, CX, SX and YS: participated in the revision of the manuscript. All authors contributed to the article and approved the submitted version.

## References

[1] Nagasawa M Spits H Ros XR. Innate lymphoid cells (ILCs): Cytokine hubs regulating immunity and tissue homeostasis. Cold Spring Harb Perspect Biol (2018) 10(12):a030304. doi: 10.1101/cshperspect.a030304 29229782 PMC6280706

[2] Panda SK Colonna M. Innate lymphoid cells in mucosal immunity. Front Immunol (2019) 10:861. doi: 10.3389/fimmu.2019.00861 31134050 PMC6515929

[3] Vivier E Artis D Colonna M Diefenbach A Di Santo JP Eberl G. Innate lymphoid cells: 10 years on. Cell (2018) 174(5):1054–66. doi: 10.1016/j.cell.2018.07.017 30142344

[4] Diefenbach A Colonna M Koyasu S. Development, differentiation, and diversity of innate lymphoid cells. Immunity (2014) 41(3):354–65. doi: 10.1016/j.immuni.2014.09.005 PMC4171710 25238093

[5] Kearley J Silver JS Sanden C Liu Z Berlin AA White N. Cigarette smoke silences innate lymphoid cell function and facilitates an exacerbated type I interleukin-33-dependent response to infection. Immunity (2015) 42(3):566–79. doi: 10.1016/j.immuni.2015.02.011 25786179

[6] Zhang Y Huang B. The development and diversity of ILCs, NK cells and their relevance in health and diseases. Adv Exp Med Biol (2017) 1024:225–44. doi: 10.1007/978-981-10-5987-2_11 28921473

[7] Gao Y Souza-Fonseca-Guimaraes F Bald T Ng SS Young A Ngiow SF. Tumor immunoevasion by the conversion of effector NK cells into type 1 innate lymphoid cells. Nat Immunol (2017) 18(9):1004–15. doi: 10.1038/ni.3800 28759001

[8] Park E Patel S Wang Q Andhey P Zaitsev K Porter S. Toxoplasma gondii infection drives conversion of NK cells into ILC1-like cells. Elife (2019) 8:e47605. doi: 10.7554/eLife.47605 31393266 PMC6703900

[9] Kabata H Moro K Koyasu S. The group 2 innate lymphoid cell (ILC2) regulatory network and its underlying mechanisms. Immunol Rev (2018) 286(1):37–52. doi: 10.1111/imr.12706 30294963

[10] van de Pavert SA Vivier E. Differentiation and function of group 3 innate lymphoid cells, from embryo to adult. Int Immunol (2016) 28(1):35–42. doi: 10.1093/intimm/dxv052 26374472

[11] Colonna M. Innate lymphoid cells: Diversity, plasticity, and unique functions in immunity. Immunity (2018) 48(6):1104–17. doi: 10.1016/j.immuni.2018.05.013 PMC6344351 29924976

[12] Konjević GM Vuletić AM Mirjačić Martinović KM Larsen AK Jurišić VB. The role of cytokines in the regulation of NK cells in the tumor environment. Cytokine (2019) 117:30–40. doi: 10.1016/j.cyto.2019.02.001 30784898

[13] Camelo A Rosignoli G Ohne Y Stewart RA Overed-Sayer C Sleeman MA. IL-33, IL-25, and TSLP induce a distinct phenotypic and activation profile in human type 2 innate lymphoid cells. Blood Adv (2017) 1(10):577–89. doi: 10.1182/bloodadvances.2016002352 PMC5728348 29296700

[14] Stanbery AG Shuchi S Jakob von M Tait Wojno ED Ziegler SF. TSLP, IL-33, and IL-25: Not just for allergy and helminth infection. J Allergy Clin Immunol (2022) 150(6):1302–13. doi: 10.1016/j.jaci.2022.07.003 PMC9742339 35863509

[15] Montaldo E Juelke K Romagnani C. Group 3 innate lymphoid cells (ILC3s): Origin, differentiation, and plasticity in humans and mice. Eur J Immunol (2015) 45(8):2171–82. doi: 10.1002/eji.201545598 26031799

[16] Dykes IM Emanueli C. Transcriptional and post-transcriptional gene regulation by long non-coding RNA. Genomics Proteomics Bioinf (2017) 15(3):177–86. doi: 10.1016/j.gpb.2016.12.005 PMC5487525 28529100

[17] Filipowicz W Bhattacharyya SN Sonenberg N. Mechanisms of post-transcriptional regulation by microRNAs: are the answers in sight? Nat Rev Genet (2008) 9(2):102–14. doi: 10.1038/nrg2290 18197166

[18] Wang J. Integrative analyses of transcriptome data reveal the mechanisms of post-transcriptional regulation. Brief Funct Genomics (2021) 20(4):207–12. doi: 10.1093/bfgp/elab004 33615339

[19] Nicolet BP Salerno F Wolkers MC. Visualizing the life of mRNA in T cells. Biochem Soc Trans (2017) 45(2):563–70. doi: 10.1042/bst20170003 28408496

[20] Yoshinaga M Takeuchi O. Post-transcriptional control of immune responses and its potential application. Clin Transl Immunol (2019) 8(6):e1063. doi: 10.1002/cti2.1063 PMC6580065 31236273

[21] Leong JW Wagner JA Ireland AR Fehniger TA. Transcriptional and post-transcriptional regulation of NK cell development and function. Clin Immunol (2017) 177:60–9. doi: 10.1016/j.clim.2016.03.003 PMC5010797 26948928

[22] Grimaldi A Pietropaolo G Stabile H Kosta A Capuano C Gismondi A. The regulatory activity of noncoding RNAs in ILCs. Cells (2021) 10(10):2742. doi: 10.3390/cells10102742 34685721 PMC8534545

[23] Kafasla P Skliris A Kontoyiannis DL. Post-transcriptional coordination of immunological responses by RNA-binding proteins. Nat Immunol (2014) 15(6):492–502. doi: 10.1038/ni.2884 24840980

[24] Zhang Y Wei Z Dong H Zhou J Yuan J Ni B. Regulation of mRNA stability by RBPs and noncoding RNAs contributing to the pathogenicity of Th17 cells. RNA Biol (2021) 18(5):647–56. doi: 10.1080/15476286.2020.1862567 PMC8078524 33302787

[25] Gordon SM Chaix J Rupp LJ Wu J Madera S Sun JC. The transcription factors T-bet and eomes control key checkpoints of natural killer cell maturation. Immunity (2012) 36(1):55–67. doi: 10.1016/j.immuni.2011.11.016 22261438 PMC3381976

[26] Bryceson YT Long EO. Line of attack: NK cell specificity and integration of signals. Curr Opin Immunol (2008) 20(3):344–52. doi: 10.1016/j.coi.2008.03.005 PMC2564616 18439809

[27] Kärre K Ljunggren HG Piontek G Kiessling R. Selective rejection of h-2-deficient lymphoma variants suggests alternative immune defence strategy. Nature (1986) 319(6055):675–8. doi: 10.1038/319675a0 3951539

[28] Hayashi H Inoue Y Tsutsui H Okamura H Nakanishi K Onozaki K. TGFbeta down-regulates IFN-gamma production in IL-18 treated NK cell line LNK5E6. Biochem Biophys Res Commun (2003) 300(4):980–5. doi: 10.1016/s0006-291x(02)02939-x 12559970

[29] Inoue Y Abe K Onozaki K Hayashi H. TGF-β decreases the stability of IL-18-induced IFN-γ mRNA through the expression of TGF-β-induced tristetraprolin in KG-1 cells. Biol Pharm Bull (2015) 38(4):536–44. doi: 10.1248/bpb.b14-00673 25832634

[30] Ye J Ortaldo JR Conlon K Winkler-Pickett R Young HA. Cellular and molecular mechanisms of IFN-gamma production induced by IL-2 and IL-12 in a human NK cell line. J Leukoc Biol (1995) 58(2):225–33. doi: 10.1002/jlb.58.2.225 7643015

[31] Hodge DL Martinez A Julias JG Taylor LS Young HA. Regulation of nuclear gamma interferon gene expression by interleukin 12 (IL-12) and IL-2 represents a novel form of posttranscriptional control. Mol Cell Biol (2002) 22(6):1742–53. doi: 10.1128/mcb.22.6.1742-1753.2002 PMC135596 11865054

[32] Ochayon DE Ali A Alarcon PC Krishnamurthy D Kottyan LC Borchers MT. IL-33 promotes type 1 cytokine expression *via* p38 MAPK in human NK cells. J Leukoc Biol (2020) 107(4):663–71. doi: 10.1002/jlb.3a0120-379rr PMC7229703 32017227

[33] Mavropoulos A Sully G Cope AP Clark AR. Stabilization of IFN-gamma mRNA by MAPK p38 in IL-12- and IL-18-stimulated human NK cells. Blood (2005) 105(1):282–8. doi: 10.1182/blood-2004-07-2782 15345584

[34] Al-Atrash G Shetty S Idell S Xue Y Kitson RP Halady PK. IL-2-mediated upregulation of uPA and uPAR in natural killer cells. Biochem Biophys Res Commun (2002) 292(1):184–9. doi: 10.1006/bbrc.2002.6627 11890690

[35] Maroof A Beattie L Zubairi S Svensson M Stager S Kaye PM. Posttranscriptional regulation of II10 gene expression allows natural killer cells to express immunoregulatory function. Immunity (2008) 29(2):295–305. doi: 10.1016/j.immuni.2008.06.012 18701085 PMC2656759

[36] Nakatsuka Y Yaku A Handa T Vandenbon A Hikichi Y Motomura Y. Profibrotic function of pulmonary group 2 innate lymphoid cells is controlled by regnase-1. Eur Respir J (2021) 57(3):2000018. doi: 10.1183/13993003.00018-2020 32978308

[37] Hikichi Y Motomura Y Takeuchi O Moro K. Posttranscriptional regulation of ILC2 homeostatic function *via* tristetraprolin. J Exp Med (2021) 218(12):e20210181. doi: 10.1084/jem.20210181 34709349 PMC8558840

[38] Zhu Y Liu Y Zhu X Wang Z Wang M. Upregulation of miR-155 regulates group 2 innate lymphoid cells by targeting c-maf in allergic rhinitis. Eur J Pharmacol (2020) 887:173564. doi: 10.1016/j.ejphar.2020.173564 32946865

[39] Zhu Y Ye F Fu Y Zhu X Wang Z Wu S. MicroRNA-155-5p regulates the Th1/Th2 cytokines expression and the apoptosis of group 2 innate lymphoid cells *via* targeting TP53INP1 in allergic rhinitis. Int Immunopharmacol (2021) 101(Pt B):108317. doi: 10.1016/j.intimp.2021.108317 34731784

[40] Liu B Liu N Zhu X Yang L Ye B Li H. Circular RNA circZbtb20 maintains ILC3 homeostasis and function *via* Alkbh5-dependent m(6)A demethylation of Nr4a1 mRNA. Cell Mol Immunol (2021) 18(6):1412–24. doi: 10.1038/s41423-021-00680-1 PMC8166869 33911218

[41] Lai HC Chang CJ Lin CS Wu TR Hsu YJ Wu TS. NK cell-derived IFN-γ protects against nontuberculous mycobacterial lung infection. J Immunol (2018) 201(5):1478–90. doi: 10.4049/jimmunol.1800123 30061197

[42] Mah AY Cooper MA. Metabolic regulation of natural killer cell IFN-γ production. Crit Rev Immunol (2016) 36(2):131–47. doi: 10.1615/CritRevImmunol.2016017387 PMC5335907 27910764

[43] Takeda K Nakayama M Hayakawa Y Kojima Y Ikeda H Imai N. IFN-γ is required for cytotoxic T cell-dependent cancer genome immunoediting. Nat Commun (2017) 8:14607. doi: 10.1038/ncomms14607 28233863 PMC5333095

[44] Ikeda H Old LJ Schreiber RD. The roles of IFN gamma in protection against tumor development and cancer immunoediting. Cytokine Growth Factor Rev (2002) 13(2):95–109. doi: 10.1016/s1359-6101(01)00038-7 11900986

[45] Ogawa K Chen F Kim YJ Chen Y. Transcriptional regulation of tristetraprolin by transforming growth factor-beta in human T cells. J Biol Chem (2003) 278(32):30373–81. doi: 10.1074/jbc.M304856200 12754205

[46] Tiedje C Holtmann H Gaestel M. The role of mammalian MAPK signaling in regulation of cytokine mRNA stability and translation. J Interferon Cytokine Res (2014) 34(4):220–32. doi: 10.1089/jir.2013.0146 24697200

[47] Briata P Forcales SV Ponassi M Corte G Chen CY Karin M. p38-dependent phosphorylation of the mRNA decay-promoting factor KSRP controls the stability of select myogenic transcripts. Mol Cell (2005) 20(6):891–903. doi: 10.1016/j.molcel.2005.10.021 16364914

[48] Brook M Tchen CR Santalucia T McIlrath J Arthur JS Saklatvala J. Posttranslational regulation of tristetraprolin subcellular localization and protein stability by p38 mitogen-activated protein kinase and extracellular signal-regulated kinase pathways. Mol Cell Biol (2006) 26(6):2408–18. doi: 10.1128/mcb.26.6.2408-2418.2006 PMC1430283 16508015

[49] Tian B Manley JL. Alternative polyadenylation of mRNA precursors. Nat Rev Mol Cell Biol (2017) 18(1):18–30. doi: 10.1038/nrm.2016.116 27677860 PMC5483950

[50] Burleson SCM Fick RB Mannie MD Olmstead SG Van Scott MR. Chapter 35 - the immune basis of allergic lung disease. In: Parent RA, editor. Comparative biology of the normal lung (Second edition). San Diego: Academic Press (2015). doi: 10.1016/B978-0-12-404577-4.00035-7

[51] Dembic Z. Chapter 6 - cytokines of the immune system: Interleukins. In: Dembic Z, editor. The cytokines of the immune system. Amsterdam: Academic Press (2015). doi: 10.1016/B978-0-12-419998-9.00006-7

[52] Dean JL Sarsfield SJ Tsounakou E Saklatvala J. p38 mitogen-activated protein kinase stabilizes mRNAs that contain cyclooxygenase-2 and tumor necrosis factor AU-rich elements by inhibiting deadenylation. J Biol Chem (2003) 278(41):39470–6. doi: 10.1074/jbc.M306345200 12882963

[53] Clark AR Dean JL Saklatvala J. Post-transcriptional regulation of gene expression by mitogen-activated protein kinase p38. FEBS Lett (2003) 546(1):37–44. doi: 10.1016/s0014-5793(03)00439-3 12829234

[54] Frevel MA Bakheet T Silva AM Hissong JG Khabar KS Williams BR. p38 mitogen-activated protein kinase-dependent and -independent signaling of mRNA stability of AU-rich element-containing transcripts. Mol Cell Biol (2003) 23(2):425–36. doi: 10.1128/mcb.23.2.425-436.2003 PMC151534 12509443

[55] Cayrol C Girard JP. Interleukin-33 (IL-33): A nuclear cytokine from the IL-1 family. Immunol Rev (2018) 281(1):154–68. doi: 10.1111/imr.12619 29247993

[56] Drube S Kraft F Dudeck J Müller AL Weber F Göpfert C. MK2/3 are pivotal for IL-33-Induced and mast cell-dependent leukocyte recruitment and the resulting skin inflammation. J Immunol (2016) 197(9):3662–8. doi: 10.4049/jimmunol.1600658 27694493

[57] Göpfert C Andreas N Weber F Häfner N Yakovleva T Gaestel M. The p38-MK2/3 module is critical for IL-33-Induced signaling and cytokine production in dendritic cells. J Immunol (2018) 200(3):1198–206. doi: 10.4049/jimmunol.1700727 29288203

[58] Clerk A Harrison JG Long CS Sugden PH. Pro-inflammatory cytokines stimulate mitogen-activated protein kinase subfamilies, increase phosphorylation of c-jun and ATF2 and upregulate c-jun protein in neonatal rat ventricular myocytes. J Mol Cell Cardiol (1999) 31(12):2087–99. doi: 10.1006/jmcc.1999.1040 10640438

[59] Oh SA Li MO. TGF-β: guardian of T cell function. J Immunol (2013) 191(8):3973–9. doi: 10.4049/jimmunol.1301843 PMC3856438 24098055

[60] Dean JL Wait R Mahtani KR Sully G Clark AR Saklatvala J. The 3' untranslated region of tumor necrosis factor alpha mRNA is a target of the mRNA-stabilizing factor HuR. Mol Cell Biol (2001) 21(3):721–30. doi: 10.1128/mcb.21.3.721-730.2001 PMC86664 11154260

[61] Alford KA Glennie S Turrell BR Rawlinson L Saklatvala J Dean JL. Heat shock protein 27 functions in inflammatory gene expression and transforming growth factor-beta-activated kinase-1 (TAK1)-mediated signaling. J Biol Chem (2007) 282(9):6232–41. doi: 10.1074/jbc.M610987200 17202147

[62] Sun L Stoecklin G Van Way S Hinkovska-Galcheva V Guo RF Anderson P. Tristetraprolin (TTP)-14-3-3 complex formation protects TTP from dephosphorylation by protein phosphatase 2a and stabilizes tumor necrosis factor-alpha mRNA. J Biol Chem (2007) 282(6):3766–77. doi: 10.1074/jbc.M607347200 17170118

[63] Marchese FP Aubareda A Tudor C Saklatvala J Clark AR Dean JL. MAPKAP kinase 2 blocks tristetraprolin-directed mRNA decay by inhibiting CAF1 deadenylase recruitment. J Biol Chem (2010) 285(36):27590–600. doi: 10.1074/jbc.M110.136473 PMC2934626 20595389

[64] Bernstein P Ross J. Poly(A), poly(A) binding protein and the regulation of mRNA stability. Trends Biochem Sci (1989) 14(9):373–7. doi: 10.1016/0968-0004(89)90011-x 2688202

[65] Peltz SW Jacobson A. mRNA stability: in trans-it. Curr Opin Cell Biol (1992) 4(6):979–83. doi: 10.1016/0955-0674(92)90129-z 1485969

[66] Sachs A. The role of poly(A) in the translation and stability of mRNA. Curr Opin Cell Biol (1990) 2(6):1092–8. doi: 10.1016/0955-0674(90)90161-7 2099802

[67] Sachs AB. Messenger RNA degradation in eukaryotes. Cell (1993) 74(3):413–21. doi: 10.1016/0092-8674(93)80043-e 7688664

[68] MacMorris MA Zorio DA Blumenthal T. An exon that prevents transport of a mature mRNA. Proc Natl Acad Sci U.S.A. (1999) 96(7):3813–8. doi: 10.1073/pnas.96.7.3813 PMC22377 10097120

[69] Tone M Powell MJ Tone Y Thompson SA Waldmann H. IL-10 gene expression is controlled by the transcription factors Sp1 and Sp3. J Immunol (2000) 165(1):286–91. doi: 10.4049/jimmunol.165.1.286 10861063

[70] Samarasinghe R Tailor P Tamura T Kaisho T Akira S Ozato K. Induction of an anti-inflammatory cytokine, IL-10, in dendritic cells after toll-like receptor signaling. J Interferon Cytokine Res (2006) 26(12):893–900. doi: 10.1089/jir.2006.26.893 17238832

[71] Saraiva M O'Garra A. The regulation of IL-10 production by immune cells. Nat Rev Immunol (2010) 10(3):170–81. doi: 10.1038/nri2711 20154735

[72] Murphy ML Wille U Villegas EN Hunter CA Farrell JP. IL-10 mediates susceptibility to leishmania donovani infection. Eur J Immunol (2001) 31(10):2848–56. doi: 10.1002/1521-4141(2001010)31:10<2848::aid-immu2848>3.0.co;2-t 11592059

[73] Bodas M Jain N Awasthi A Martin S Penke Loka RK Dandekar D. Inhibition of IL-2 induced IL-10 production as a principle of phase-specific immunotherapy. J Immunol (2006) 177(7):4636–43. doi: 10.4049/jimmunol.177.7.4636 16982902

[74] Powell MJ Thompson SA Tone Y Waldmann H Tone M. Posttranscriptional regulation of IL-10 gene expression through sequences in the 3'-untranslated region. J Immunol (2000) 165(1):292–6. doi: 10.4049/jimmunol.165.1.292 10861064

[75] Ernst O Glucksam-Galnoy Y Bhatta B Athamna M Ben-Dror I Glick Y. Exclusive temporal stimulation of IL-10 expression in LPS-stimulated mouse macrophages by cAMP inducers and type I interferons. Front Immunol (2019) 10:1788. doi: 10.3389/fimmu.2019.01788 31447835 PMC6691811

[76] Jagiello I Beullens M Vulsteke V Wera S Sohlberg B Stalmans W. NIPP-1, a nuclear inhibitory subunit of protein phosphatase-1, has RNA-binding properties. J Biol Chem (1997) 272(35):22067–71. doi: 10.1074/jbc.272.35.22067 9268347

[77] Myer VE Fan XC Steitz JA. Identification of HuR as a protein implicated in AUUUA-mediated mRNA decay. EMBO J (1997) 16(8):2130–9. doi: 10.1093/emboj/16.8.2130 PMC1169815 9155038

[78] Lewis T Gueydan C Huez G Toulmé JJ Kruys V. Mapping of a minimal AU-rich sequence required for lipopolysaccharide-induced binding of a 55-kDa protein on tumor necrosis factor-alpha mRNA. J Biol Chem (1998) 273(22):13781–6. doi: 10.1074/jbc.273.22.13781 9593721

[79] Sakai K Kitagawa Y Hirose G. Binding of neuronal ELAV-like proteins to the uridine-rich sequence in the 3'-untranslated region of tumor necrosis factor-alpha messenger RNA. FEBS Lett (1999) 446(1):157–62. doi: 10.1016/s0014-5793(99)00206-9 10100634

[80] Gueydan C Droogmans L Chalon P Huez G Caput D Kruys V. Identification of TIAR as a protein binding to the translational regulatory AU-rich element of tumor necrosis factor alpha mRNA. J Biol Chem (1999) 274(4):2322–6. doi: 10.1074/jbc.274.4.2322 9890998

[81] Jarrousse AS Petit F Kreutzer-Schmid C Gaedigk R Schmid HP. Possible involvement of proteasomes (prosomes) in AUUUA-mediated mRNA decay. J Biol Chem (1999) 274(9):5925–30. doi: 10.1074/jbc.274.9.5925 10026217

[82] Owen CA Campbell EJ. The cell biology of leukocyte-mediated proteolysis. J Leukoc Biol (1999) 65(2):137–50. doi: 10.1002/jlb.65.2.137 10088596

[83] Vaday GG Lider O. Extracellular matrix moieties, cytokines, and enzymes: dynamic effects on immune cell behavior and inflammation. J Leukoc Biol (2000) 67(2):149–59. doi: 10.1002/jlb.67.2.149 10670574

[84] Albertsson P Kim MH Jonges LE Kitson RP Kuppen PJ Johansson BR. Matrix metalloproteinases of human NK cells. In Vivo (2000) 14(1):269–76. 10757086

[85] G. al-Atrash RP Kitson YX R. H. G. Cooperation of urokinase plasminogen activator and matrix metalloproteinases in NK cell invasion. In Vivo (2000) 14(5):565–70. 11125540

[86] Shetty S Idell S. Post-transcriptional regulation of urokinase mRNA. identification of a novel urokinase mRNA-binding protein in human lung epithelial cells *in vitro* . J Biol Chem (2000) 275(18):13771–9. doi: 10.1074/jbc.275.18.13771 10788498

[87] Shetty S Idell S. A urokinase receptor mRNA binding protein-mRNA interaction regulates receptor expression and function in human pleural mesothelioma cells. Arch Biochem Biophys (1998) 356(2):265–79. doi: 10.1006/abbi.1998.0789 9705217

[88] Shetty S Idell S. A urokinase receptor mRNA binding protein from rabbit lung fibroblasts and mesothelial cells. Am J Physiol (1998) 274(6):L871–82. doi: 10.1152/ajplung.1998.274.6.L871 9609725

[89] Shetty S Kumar A Idell S. Posttranscriptional regulation of urokinase receptor mRNA: identification of a novel urokinase receptor mRNA binding protein in human mesothelioma cells. Mol Cell Biol (1997) 17(3):1075–83. doi: 10.1128/mcb.17.3.1075 PMC231832 9032234

[90] Timonen T Helander TS. Natural killer cell-target cell interactions. Curr Opin Cell Biol (1997) 9(5):667–73. doi: 10.1016/s0955-0674(97)80120-2 9330870

[91] Timonen T. Natural killer cells: endothelial interactions, migration, and target cell recognition. J Leukoc Biol (1997) 62(6):693–701. doi: 10.1002/jlb.62.6.693 9400809

[92] Uchiyama A Morisaki T Torisu M. Evidence that induction and regulation of lymphokine-activated killer (LAK) activity are mediated by changes in tumour-binding potential of lymphocytes after activation by interleukin-2 (IL-2). Immunology (1991) 74(1):94–8. PMC1384677 1657765

[93] Quillet-Mary A Cavarec L Kermarrec N Marchiol-Fournigault C Gil ML Conjeaud H. Target lysis by human LAK cells is critically dependent upon target binding properties, but LFA-1, LFA-3 and ICAM-1 are not the major adhesion ligands on targets. Int J Cancer (1991) 47(3):473–9. doi: 10.1002/ijc.2910470328 1704356

[94] Moro K Yamada T Tanabe M Takeuchi T Ikawa T Kawamoto H. Innate production of T(H)2 cytokines by adipose tissue-associated c-Kit(+)Sca-1(+) lymphoid cells. Nature (2010) 463(7280):540–4. doi: 10.1038/nature08636 20023630

[95] Price AE Liang HE Sullivan BM Reinhardt RL Eisley CJ Erle DJ. Systemically dispersed innate IL-13-expressing cells in type 2 immunity. Proc Natl Acad Sci U.S.A. (2010) 107(25):11489–94. doi: 10.1073/pnas.1003988107 PMC2895098 20534524

[96] Starkey MR McKenzie AN Belz GT Hansbro PM. Pulmonary group 2 innate lymphoid cells: surprises and challenges. Mucosal Immunol (2019) 12(2):299–311. doi: 10.1038/s41385-018-0130-4 30664706 PMC6436699

[97] Hams E Armstrong ME Barlow JL Saunders SP Schwartz C Cooke G. IL-25 and type 2 innate lymphoid cells induce pulmonary fibrosis. Proc Natl Acad Sci U.S.A. (2014) 111(1):367–72. doi: 10.1073/pnas.1315854111 PMC3890791 24344271

[98] Li D Guabiraba R Besnard AG Komai-Koma M Jabir MS Zhang L. IL-33 promotes ST2-dependent lung fibrosis by the induction of alternatively activated macrophages and innate lymphoid cells in mice. J Allergy Clin Immunol (2014) 134(6):1422–32.e11. doi: 10.1016/j.jaci.2014.05.011 24985397 PMC4258609

[99] Kindermann M Knipfer L Atreya I Wirtz S. ILC2s in infectious diseases and organ-specific fibrosis. Semin Immunopathol (2018) 40(4):379–92. doi: 10.1007/s00281-018-0677-x 29623414

[100] Mizgalska D Wegrzyn P Murzyn K Kasza A Koj A Jura J. Interleukin-1-inducible MCPIP protein has structural and functional properties of RNase and participates in degradation of IL-1beta mRNA. FEBS J (2009) 276(24):7386–99. doi: 10.1111/j.1742-4658.2009.07452.x 19909337

[101] Matsushita K Takeuchi O Standley DM Kumagai Y Kawagoe T Miyake T. Zc3h12a is an RNase essential for controlling immune responses by regulating mRNA decay. Nature (2009) 458(7242):1185–90. doi: 10.1038/nature07924 19322177

[102] Liu C Adamson E Mercola D. Transcription factor EGR-1 suppresses the growth and transformation of human HT-1080 fibrosarcoma cells by induction of transforming growth factor beta 1. Proc Natl Acad Sci U.S.A. (1996) 93(21):11831–6. doi: 10.1073/pnas.93.21.11831 PMC38144 8876223

[103] Liu C Calogero A Ragona G Adamson E Mercola D. EGR-1, the reluctant suppression factor: EGR-1 is known to function in the regulation of growth, differentiation, and also has significant tumor suppressor activity and a mechanism involving the induction of TGF-beta1 is postulated to account for this suppressor activity. Crit Rev Oncog (1996) 7(1-2):101–25. 9109500

[104] Maazi H Patel N Sankaranarayanan I Suzuki Y Rigas D Soroosh P. ICOS:ICOS-ligand interaction is required for type 2 innate lymphoid cell function, homeostasis, and induction of airway hyperreactivity. Immunity (2015) 42(3):538–51. doi: 10.1016/j.immuni.2015.02.007 PMC4366271 25769613

[105] Uehata T Iwasaki H Vandenbon A Matsushita K Hernandez-Cuellar E Kuniyoshi K. Malt1-induced cleavage of regnase-1 in CD4(+) helper T cells regulates immune activation. Cell (2013) 153(5):1036–49. doi: 10.1016/j.cell.2013.04.034 23706741

[106] Saini Y Chen J Patial S. The tristetraprolin family of RNA-binding proteins in cancer: Progress and future prospects. Cancers (Basel) (2020) 12(6):1539. doi: 10.3390/cancers12061539 32545247 PMC7352335

[107] Lykke-Andersen J Wagner E. Recruitment and activation of mRNA decay enzymes by two ARE-mediated decay activation domains in the proteins TTP and BRF-1. Genes Dev (2005) 19(3):351–61. doi: 10.1101/gad.1282305 PMC546513 15687258

[108] Lai WS Carballo E Thorn JM Kennington EA Blackshear PJ. Interactions of CCCH zinc finger proteins with mRNA. binding of tristetraprolin-related zinc finger proteins to au-rich elements and destabilization of mRNA. J Biol Chem (2000) 275(23):17827–37. doi: 10.1074/jbc.M001696200 10751406

[109] Lai WS Stumpo DJ Wells ML Gruzdev A Hicks SN Nicholson CO. Importance of the conserved carboxyl-terminal CNOT1 binding domain to tristetraprolin activity in vivo. Mol Cell Biol (2019) 39(13):e00029-19. doi: 10.1128/mcb.00029-19 31036567 PMC6580703

[110] Nussbaum JC Van Dyken SJ von Moltke J Cheng LE Mohapatra A Molofsky AB. Type 2 innate lymphoid cells control eosinophil homeostasis. Nature (2013) 502(7470):245–8. doi: 10.1038/nature12526 PMC3795960 24037376

[111] Chu VT Beller A Rausch S Strandmark J Zänker M Arbach O. Eosinophils promote generation and maintenance of immunoglobulin-a-expressing plasma cells and contribute to gut immune homeostasis. Immunity (2014) 40(4):582–93. doi: 10.1016/j.immuni.2014.02.014 24745334

[112] Jung Y Wen T Mingler MK Caldwell JM Wang YH Chaplin DD. IL-1β in eosinophil-mediated small intestinal homeostasis and IgA production. Mucosal Immunol (2015) 8(4):930–42. doi: 10.1038/mi.2014.123 PMC4481137 25563499

[113] O'Connell RM Rao DS Chaudhuri AA Baltimore D. Physiological and pathological roles for microRNAs in the immune system. Nat Rev Immunol (2010) 10(2):111–22. doi: 10.1038/nri2708 20098459

[114] Gregory RI Chendrimada TP Cooch N Shiekhattar R. Human RISC couples microRNA biogenesis and posttranscriptional gene silencing. Cell (2005) 123(4):631–40. doi: 10.1016/j.cell.2005.10.022 16271387

[115] O'Brien J Hayder H Zayed Y Peng C. Overview of MicroRNA biogenesis, mechanisms of actions, and circulation. Front Endocrinol (Lausanne) (2018) 9:402. doi: 10.3389/fendo.2018.00402 30123182 PMC6085463

[116] Macfarlane LA Murphy PR. MicroRNA: Biogenesis, function and role in cancer. Curr Genomics (2010) 11(7):537–61. doi: 10.2174/138920210793175895 PMC3048316 21532838

[117] Conejo-Garcia JR Rodriguez PC. C-maf: a bad influence in the education of macrophages. J Clin Invest (2020) 130(4):1629–31. doi: 10.1172/jci135444 PMC7108897 32175921

[118] Castleman MJ Dillon SM Purba CM Cogswell AC Kibbie JJ McCarter MD. Commensal and pathogenic bacteria indirectly induce IL-22 but not IFNγ production from human colonic ILC3s *via* multiple mechanisms. Front Immunol (2019) 10:649. doi: 10.3389/fimmu.2019.00649 30984202 PMC6450192

[119] Zhao X Cai Y Xu J. Circular RNAs: Biogenesis, mechanism, and function in human cancers. Int J Mol Sci (2019) 20(16):3926. doi: 10.3390/ijms20163926 31412535 PMC6720291

[120] Liu B Ye B Zhu X Yang L Li H Liu N. An inducible circular RNA circKcnt2 inhibits ILC3 activation to facilitate colitis resolution. Nat Commun (2020) 11(1):4076. doi: 10.1038/s41467-020-17944-5 32796851 PMC7427797

[121] Liu N He J Fan D Gu Y Wang J Li H. Circular RNA circTmem241 drives group III innate lymphoid cell differentiation *via* initiation of Elk3 transcription. Nat Commun (2022) 13(1):4711. doi: 10.1038/s41467-022-32322-z 35953472 PMC9372085

[122] Zhang S Zhao BS Zhou A Lin K Zheng S Lu Z. m(6)A demethylase ALKBH5 maintains tumorigenicity of glioblastoma stem-like cells by sustaining FOXM1 expression and cell proliferation program. Cancer Cell (2017) 31(4):591–606.e6. doi: 10.1016/j.ccell.2017.02.013 28344040 PMC5427719

[123] Liu J Yue Y Han D Wang X Fu Y Zhang L. A METTL3-METTL14 complex mediates mammalian nuclear RNA N6-adenosine methylation. Nat Chem Biol (2014) 10(2):93–5. doi: 10.1038/nchembio.1432 PMC3911877 24316715

[124] Badrani JH Strohm AN Lacasa L Civello B Cavagnero K Haung YA. RNA-Binding protein RBM3 intrinsically suppresses lung innate lymphoid cell activation and inflammation partially through CysLT1R. Nat Commun (2022) 13(1):4435. doi: 10.1038/s41467-022-32176-5 35908044 PMC9338970

[125] Liu S Chen L Chen H Xu K Peng X Zhang M. Circ_0119872 promotes uveal melanoma development by regulating the miR-622/G3BP1 axis and downstream signalling pathways. J Exp Clin Cancer Res (2021) 40(1):66. doi: 10.1186/s13046-021-01833-w 33579337 PMC7881613

[126] Shen W Wang C Huang B. Oxidative stress-induced circHBEGF promotes extracellular matrix production *via* regulating miR-646/EGFR in human trabecular meshwork cells. Oxid Med Cell Longev (2020) 2020:4692034. doi: 10.1155/2020/4692034 33335643 PMC7722639

[127] Saikishore R Velmurugan P Ranjithkumar D Latha R Sathiamoorthi T Arun A. The circular RNA-miRNA axis: A special RNA signature regulatory transcriptome as a potential biomarker for OSCC. Mol Ther Nucleic Acids (2020) 22:352–61. doi: 10.1016/j.omtn.2020.09.001 PMC7530261 33230440

